# The influence of anesthesia type on perioperative maternal glycemic-stress response during elective cesarean section: A prospective cohort study

**DOI:** 10.1016/j.amsu.2021.102209

**Published:** 2021-03-04

**Authors:** Diab A. Bani Hani, Omar F. Altal, Adel Bataineh, Mahmoud Al Athamneh, Mohammad Altarawneh, Moath Alshawaqfeh, Hajar Haddouchane, Sultan M. Al-Zyoud, Musa'b Hamarsheh, Ala”a A. Alhowary

**Affiliations:** a Department of Anesthesia and Recovery, Faculty of Medicine, Jordan University of Science and Technology, Irbid, 21110, Jordan; b Department of Obstetrics and Gynecology, Faculty of Medicine, Jordan University of Science and Technology, Irbid, 21110, Jordan; c Faculty of Medicine, Jordan University of Science and Technology, Irbid, 21110, Jordan; d Department of Anesthesia, Princess Badea Training Hospital, Ministry of Health, Irbid, Jordan

**Keywords:** General anesthesia, Cesarean section, Spinal anesthesia, Glucose

## Abstract

**Background:**

It has been known that the type of anesthesia can affect the stress response to surgery in form of hyperglycemia. This study aims to evaluate and compare the influence of general anesthesia (GA) and spinal anesthesia (SA) on pregnant women who are scheduled to undergo cesarean section (CS) and to understand the impact of obstetrical factors on the maternal hyperglycemic-stress response during CS.

**Methods:**

Prospectively, we identified, assessed and followed those pregnant women who were scheduled to undergo elective CS surgery. The included group comprises any women who was scheduled to undergo an elective CS. The preoperative and postoperative blood glucose levels were measured and compared between both groups (GA and SA groups).

**Results:**

The study included 302 patients who satisfied the inclusion and exclusion criteria of the study. GA was more commonly utilized in cesarean sections (56.6%) compared with SA (43.4%). The average gestational age at time of delivery was 37.4 weeks. The post-operative readings were significantly higher in the GA group with a mean sugar level of 110.1 mg/dL and a mean sugar level in the SA group of 87.7 mg/dL (P = 0.00). After performing multiple regression analysis, it was revealed that the type of anesthesia is the most independent factor affecting the postoperative sugar level.

**Conclusion:**

GA causes higher blood glucose concentrations than SA, which indicates that the impact of GA on hormonal stress response and metabolic hemostasis is greater than in SA.

## Highlights

• The type of anesthesia has an influence on hyperglycemic response to surgery.
• The impact of GA on plasma glucose levels was substantially more than the SA.
• The hormonal stress response is significantly more in GA.

## 1 Introduction

Many efforts were elaborated to investigate the potential beneficial effects of preservation of glucose homeostasis and early avoidance of stress-induced hyperglycemia in surgical patients. Acute hyperglycemia can be developed as a part of the metabolic response to the stressful conditions such as surgery. This response can significantly compromise the immunological system and leads to poor clinical outcome [[1], [2], [3], [4]]. The degree of this response was related proportionally to the severity and length of the surgical injury [5], and the level of insulin resistance [6].

Normal pregnancy is characterized by a number of maternal metabolic alternations in order to meet the energy requirements of the growing fetus which is most evident in the third trimester. A relative hypoglycemia is observed during the postabsorptive period despite an elevated plasma insulin concentration and an enhanced hepatic glucose production. In addition, the glucose utilization rate by peripheral maternal tissues is lowered in late gestation indicating that the mother supplies glucose to the fetus at the expense of her own tissues [7].

It is well-known that the type of anesthetic technique has an influence on hyperglycemic response to surgery [8]. During surgery, inhalation anesthesia has more pronounced stressful hyperglycemia. In this study, we aim to investigate the effect of general anesthesia (GA) and spinal anesthesia (SA) on pregnant women who are scheduled to undergo cesarean section (CS). Also, this study aims to understand the effect of other factors on the maternal stress response during CS.

## 2 Material and methods

This study was conducted at King Abdullah University Hospital, a tertiary care center that is affiliated with the Jordan University of Science and Technology, and Princess Badi'ah Hospital that is affiliated to the Ministry of Health. After obtaining the institutional review approval, we prospectively identified, assessed and followed those pregnant women who were scheduled to undergo elective CS surgery between June 2019 and December 2019. The following information were obtained: demographics (e.g. age), obstetrical history (gestational age at delivery, gravidity, parity, diabetes, number of gestations, steroid injections, and the utilized medications in pregnancy), and the presence of third trimester-urinary tract infection. In addition, the type of anesthesia was determined. Moreover, the preoperative and postoperative blood glucose levels were measured.

The included group comprises any women who was scheduled to undergo an elective CS with the grades 1 and 2 physical status according to American Society of Anesthesiologists, above the age of 18 years, and fasting time preoperatively more than 8 h. Women diagnosed with diabetes mellitus, chronic advanced renal disease, and preeclamptic disorders were excluded. In addition, all cases of SA who was converted to GA were excluded. Moreover, any women with absolute contraindications for either the GA or SA were also excluded. Women with gestational diabetes were included in the study to investigate the impact of gestational diabetes. Based on their choices, pregnant women were categorized into two groups: the GA group and SA group. Written informed consent was obtained from all women.

### 2.1 Anesthetic setting

Consultant anesthesiologists and senior residents carried out and supervised the conduction of anesthesia. At the theater, two intravenous cannulas were inserted, and continuous monitoring of blood pressure, oxygen saturation, respiratory rate, and electrocardiogram were conducted.

#### 2.1.1 Spinal anesthesia

At the level of L3-L4 or L4-L5 of the vertebral column, the SA was conducted under aseptic technique using 25- or 27-gauge spinal needles with 2.3 ml of 0.5% heavy bupivacaine and 0.4 ml of 0.005% fentanyl. Adequate hydration with crystalloid solution and was performed before the procedure along with100% O2 through a nasal cannula.

#### 2.1.2 General anesthesia

Rapid-sequence induction was done with the insertion of endotracheal tube sized 6.5–7.5 mm; the induction was performed by using propofol and rocuronium. Anesthesia was maintained using isoflurane in 50% oxygen and 50% air. After delivery of the baby and cutting the umbilical cord, fentanyl was given, inhaled anesthetic agents were discontinued and anesthesia was maintained with a propofol infusion. At the end of surgery, neostigmine and atropine were given intravenously.

#### 2.1.3 Obstetrical settings

The CS operations were performed by consultant obstetricians. Foley's catheter was inserted. Lower-segment transverse uterine incision was carried out.

After delivery of the baby, all women received intravenous 10 IU oxytocin bolus and 20 IU oxytocin infusion over 1 h. Both groups were given 2–3 L of crystalloids fluid.

#### 2.1.4 Glucose measurement

For the women underwent GA, the blood glucose concentration was obtained 5 min before induction of anesthesia and 5 min after the surgery. For the SA group, the glucose concentration was obtained 5 min before the injection of the local anesthetia and 5 min after the surgery.

### 2.2 Statistical analysis

Statistical analyses were performed using IBM SPSS Statistics Software (v.21), 2012. The factors that were examined relating to glucose level were described using the frequency distribution of categorical variables and the mean ± standard error (SE) for continuous variables. The data were examined using Pearson's chi-square (χ2) tests to examine associations between categorical variables and Student's t-tests were used for continuous variables. A value of p < 0.05 indicated a statistically significant difference. Multiple linear regression analysis was utilized to examine the effect of many factors on the glucose response.

## 3 Results

### 3.1 Patients characteristics

The study included 302 patients who satisfied the inclusion and exclusion criteria of the study. The average age of mothers included in the study was 31 years (Table 1), the majority of patients in our sample were Jordanian (95.7%). The number of patients with gestational diabetes was 14 with a percentage of 4.6%.

**Table 1:** General characteristics.

Variable	Number	Percent (%)
	Mean ± SE	
**Mother age (years)**	31.0 ± .3	
**Gestational diabetes**	14	4.6
**Steroid Injection**	111	36.8
**Gravidity**		
1	39	12.9
2	66	21.9
3	61	20.2
4	56	18.5
5	37	12.3
6	15	5.0
7	8	2.6
8	12	4.0
9	6	2.0
11	1	.3
13	1	.3
**Parity**		
0	51	16.9
1	80	26.5
2	62	20.5
3	55	18.2
4	33	10.9
5	11	3.6
6	5	1.7
7	4	1.3
8	1	.3
**Number of Miscarriages**		
1	69	67.0
2	21	20.4
3	8	7.8
4	3	2.9
6	1	1
8	1	1
**GBS**	16	5.3
**Nationality**		
Jordanian	289	95.7
Non- Jordanian	13	4.3
**Number of fetus**		
1	290	96.0
2	8	2.6
3	4	1.3
**Type of anesthesia**		
GA	171	56.6
SA	131	43.4
**Gestational age (weeks)**	37.4 ± .1	
**Gestational age (days)**	263.3 ± .7	
**Birth weight (gram)**	3008.5 ± 32.0	
**Mother Sugar before delivery (mg/dL)**	93.4 ± 1.1	
**Mother sugar after delivery (mg/dL)**	100.3 ± 1.2	

Abbreviations: SE: standard error; GBS: group-B streptococcus; GA: gestational age; SA: spinal anesthesia.

About 36.8% of patients in our sample had received a steroid injection before delivery. As regards to gravidity and parity, gravida 2 (21.9%) and para 1 (26.5%) were most commonly encountered. Also, most women (67%) has a history of single miscarriage. In addition, 5.3% of the women had Group B streptococcus positive colonization or had a genitourinary infection of another cause. Most patients had singleton pregnancy with a percentage of (96%).

GA was more commonly utilized in cesarean sections (56.6%) compared with SA (43.4%). The average gestational age at time of delivery was 37.4 weeks and the average birthweight of neonates was 3008.5 g. The average reading of maternal glucose before delivery was 93.4 mg/dL compared to 100.3 mg/dL after delivery.

### 3.2 Differences GA and SA

It was found that the age of mothers is similar between both groups. However, the results revealed that mothers who had SA delivered at earlier gestational ages with a mean gestational age for the SA group of 36.7 weeks and for the GA group of 37.9 weeks (P = 0.000) (Table 2).

**Table 2:** GA versus SA.

Variable	GA N (% within GA)	Spinal N (% within spinal)	p- Value
**Mother age (years) (mean** ± **SE)**	30.5 ± .5	31.5 ± .5	NS
**Gestational age (weeks) (mean** ± **SE)**	37.9 ± .1	36.7 ± .2	0.0
**Gestational age (Days)**	266 ± .9	260.0 ± 1.1	0.0
**Birth weight (gram) (mean** ± **SE)**	3069 ± 40.4	2931.8 ± 50.7	0.033
**Mother's sugar before delivery (mg/dL) (mean** ± **SE)**	100.8 ± 1.4	83.6 ± 1.5	0.0
**Mother's sugar after delivery (mg/dL) (mean** ± **SE)**	110.1 ± 1.6	87.7 ± 1.3	0.0
**Mother's perioperative difference in sugar level (mg/dL) (mean** ± **SE)**	9.1 ± 1.6	4.2 ± 1.3	0.02
**Gestational diabetes**	6 (3.5)	8 (6.1)	NS
**Gravidity**			
1	22 (12.9)	17 (13.0)	NS
2	37 (21.6)	29 (22.1)	
3	32 (18.7)	29 (22.1)	
4	32 (18.7)	24 (18.3)	
5	25 (14.6)	12 (9.2)	
6	6 (3.5)	9 (6.9)	
7	4 (2.3)	4 (3.1)	
8	9 (5.3)	3 (2.3)	
9	3 (1.8)	3 (2.3)	
11	0 (0.0)	1 (0.8)	
13	1 (0.6)	0 (0.0)	
**GBS/GUI**	2 (1.2)	14 (10.7)	0.0
**Number of babies**			
1	168 (98.2)	122 (93.1)	NS
2	2 (1.2)	6 (4.6)	
3	1 (0.6)	3 (2.3)	
**Steroid Injection**	28 (16.4)	83 (63.4)	0.0

Abbreviations: SE: standard error; GBS: group-B streptococcus; GA: gestational age; SA: spinal anesthesia.

Regarding pre-operative maternal blood sugar, the readings were significantly higher in women who underwent general anesthesia 100.8 mg/dL in comparison with women who underwent spinal anesthesia 83.6 ± 1.5 (P = 0.00). Also, the post-operative readings were significantly higher in the GA group with a mean sugar level of 110.1 mg/dL and a mean sugar level in the SA group of 87.7 mg/dL (P = 0.00). Moreover, the perioperative difference in the sugar level (postoperative-preoperative) was significantly higher in the GA group Table 2.

There was no significant difference in the number of gestational diabetes patients between both groups. The gravidity and parity did not affect the type of the utilized anesthesia. Most patients who has GBS colonization or a history of GUI underwent SA (10.7%) compared to 1.2% who underwent GA (P = 0.00). The number of fetus did not affect the type of anesthesia used. Higher number of patients who had a steroid injection before delivery underwent SA (63.4%) compared to 16.4% in the GA group (P = 0.00).

### 3.3 Factors affecting pre-operative and post-operative maternal blood sugar

The maternal blood sugar was significantly associated with receiving a steroid injection, type of anesthesia, and the gestational age (Table 3).

**Table 3:** Factors affecting the pre- and post-operative maternal glucose response.

Factors	Pre- operative maternal blood sugar (Mean ± S.E); B value for interval variable	P - value	Post- operative maternal blood sugar (Mean ± S.E); B value for interval variable	p- value
**Steroid Injection**				
Yes	85.6 ± 1.6	0.000	91.2 ± 1.6	0.000
No	97.9 ± 1.4		105.7 ± 1.6	
**Gravidity**				
1	95.9 ± 3.7	NS	104.2 ± 2.9	NS
2	93.0 ± 2.1		99.9 ± 2.5	
3	91.0 ± 1.9		98.9 ± 3.1	
4	92.7 ± 2.8		100.1 ± 3.0	
5	99.0 ± 3.8		105.2 ± 3.8	
6	89.3 ± 5.9		95.9 ± 6.7	
7	88.6 ± 6.7		99.6 ± 7.8	
8	91.3 ± 5.4		90.7 ± 3.8	
9	97.5 ± 6.6		97.7 ± 8.5	
11	81.0 ± 0.0		71.0 ± 0.0	
13	111.0 ± 0.0		107.0 ± 0.0	
**Parity**				
0	97.7 ± 3.4	NS	104.2 ± 2.7	NS
1	94.2 ± 2.2		101.2 ± 2.6	
2	89.0 ± 1.9		96.8 ± 2.8	
3	92.4 ± 2.3		101.2 ± 2.8	
4	93.2 ± 4.1		96.5 ± 3.8	
5	96.8 ± 5.4		103.7 ± 9.2	
6	102.0 ± 5.8		107.0 6.8	
7	88.8 ± 2.6		93.0 ± 6.1	
8	75.0 ± 0.0		70.0 ± 0.0	
**Miscarriages**				
1	94.1 ± 2.0	NS	101.8 ± 3.0	NS
2	98.2 ± 6.4		101.7 ± 4.6	
3	97.3 ± 4.5		96.3 ± 6.4	
4	97.0 ± 34.4		89.0 ± 9.7	
6	81.0 ± 0.0		71.0 ± 0.0	
8	111.0 ± 0.0		107.0 ± 0.0	
**GBS**				
No	93.7 ± 1.2	NS	100.8 ± 1.3	NS
Yes	87.3 ± 2.6		91.1 ± 4.1	
**Number of babies**				
1	93.4 ± 1.2	NS	100.2 ± 1.3	NS
2	94.1 ± 4.9		106.0 ± 7.1	
3	85.8 ± 6.2		96.5 ± 12.9	
**Type of anesthesia**				
GA	100.8 ± 1.4	0.000	110.1 ± 1.6	0.000
Spinal	83.6 ± 1.5		87.7 ± 1.3	
**Mother age**	- 0.3 ± 0.2	NS	−0.4 ± 0.2	NS
**Gestational age (weeks)**	1.3 ± 0.6	0.034	2.3 ± 0.7	0.001
**Gestational age (Days)**	0.1 ± 0.1	NS	0.2 ± 0.1	0.019
**Birthweight (gram)**	0.0 ± 0.0	NS	0.0 ± 0.0	NS

Abbreviations: SE: standard error; GBS: group-B streptococcus; GA: gestational age; SA: spinal anesthesia.

Receiving a steroid injection significantly lowers the maternal blood sugar (P = 0.000). Also, undergoing SA was associated with lower blood sugar readings compared with GA (P = 0.000). The gestational age calculated in both weeks and days significantly affect the maternal blood sugar; for each 1 week increase in gestational age, the pre-operative maternal blood sugar increased by 1.3 mg/dL (P = 0.034) and the post-operative blood sugar increased by 2.3 mg/dL (P = 0.001).

The gravidity, parity and number of previous miscarriages did not affect the maternal sugar reading. Also, getting a positive GBS or having a GUI did not change the readings as well. The mother age did not appear to affect pre-operative and post-operative maternal blood sugar. Furthermore, the number of babies was not significantly associated with maternal blood sugar level. After performing multiple linear regression analysis, it was found that the type of anesthesia is the most independent factor affecting the postoperative sugar level.

## 4 Discussion

In this study, we are comparing the impact of spinal and general anesthesia on blood glucose levels before and after cesarean section in non-diabetics. Despite the fact that the mean blood glucose levels were significantly increased after surgery in both groups, GA group had higher glycemic response. Therefore, SA had a lower effect on the glycemic response during cesarean section.

Turina et al. [2] illustrated that a short period of hyperglycemia is directly related to high mortality rate and susceptibility to infections in critically ill patients associated with a significant fall in monocyte HLA-DR expression because of both hyperglycemia and hyperinsulinemia.

The process of hyperglycemia management puts the patient in an increased risk of having a hypoglycemic response which will result in developing the risks associated with hypoglycemia, and thus avoidance of stress-induced hyperglycemia is preferable for treating dysglycemia [9]. Various researches on isoflurane inhalational anesthetic showed an elevation in the glucose level during anesthesia in the presence and absence of surgical stress related to impaired glucose tolerance and stimulating the production of whole-body glucose [1,3,10]. Moreover, the response of hyperglycemic stress in patients who undergo a major abdominal surgical intervention under the effect of isoflurane in general anesthesia might be responsible for an elevation in endogenous glucose production accompanied by a decline in the utilization of glucose [1,11]. It was stated in a study by Tanaka et al. [12] that during a dose-independent anesthesia using sevoflurane and isoflurane there was a glucose intolerance response and a decline in insulin secretion and glucose utilization. Cok et al. [13] stated that propofol reduced the rise in blood glucose even though isoflurane and propofol, both joint with remifentanil, provided an insulin and cortisol response to surgery in craniotomy operations that is clinically comparable. As a result, propofol may have an advantage over isoflurane when strict glycemic control is desired.

Regarding neuraxial anesthesia whether epidural or spinal using local anesthetics, it leads to a blockage of afferent input from the site of operation to the central nervous system and the hypothalamic-pituitary axis and efferent autonomic neuronal pathways to the liver and adrenal medulla. As a consequent, a significant inhibition occurs to the adrenocortical and glycemic responses to surgery [4,14]. Kehlet's study [15] revealed that epidural blockage reduced the hyperglycemic response during surgery, mostly mediated by its inhibiting effect on the hypothalamic-pituitary-adrenal axis. Various studies focusing at glucose tolerance tests during pelvic surgical procedures illustrated that epidural blockade improved tissue uptake of glucose [16,17]. By contrast, some studies showed that epidural blockade reduced the hyperglycemic response during surgery through inhibition of the release of glucose by the liver instead of improving glucose utilization by the tissues [18,19]. A study by Lattermann et al. [4] revealed that epidural blockage reduced the hyperglycemic changes caused by abdominal surgery by modifying glucose generation without intervening with the utilization of glucose. Yet, there is no clear evidence if the inhibition of the epidural blockade affecting the hyperglycemic response operatively was a result of the improvement in glucose uptake by the tissues, a decline in production of glucose, or a combined picture of both. Generally, it is known that epidural blockage using a local anesthetic prevents the endocrine and metabolic responses to surgery as well as hyperglycemia [4]. Another study by Enquist et al. [20] revealed that an epidural block, prior to surgery, inhibited the rise in blood glucose and cortisol concentrations postoperatively in patients who undergone hysterectomy. Recently, Hadimioglu et al. [21] illustrated that a combination of general and epidural anesthesia, in comparison with general anesthesia solely, attenuated inflammatory response and insulin resistance in response to the stress caused by the renal transplantation procedure and that prevention of stress responses had a positive effect on the hospitalization period after surgery. As a summary for the previously mentioned studies, epidural anesthesia attenuates the hyperglycemic response during surgery. In this study, SA had comparable results to the epidural anesthesia. Both techniques are neuraxial techniques.

Hyperglycemia in diabetic patients undergoing surgery is responsible for an elevated ratio of surgical site infections (SSI), myocardial infarction (MI), stroke, and death [[22], [23], [24], [25], [26]]. Hyperglycemia also occurs in up to 67% of surgical patients who are not established diabetics, and its impact has not been well developed in surgical patients without diabetes (NDM) [22,27]. Recently, it was demonstrated in some studies that there is a greater risk of complications related to hyperglycemia in NDM patients in comparison with DM patients. A study by Kwon et al. [28] illustrated that NDM patients who had perioperative hyperglycemia had double the risk of infections, re-operative interventions, and in-hospital deaths as DM patients and hyperglycemia. In addition, Frisch et al. [29] reported an elevated risk of 30-day death caused by hyperglycemia for NDM patients in comparison with diabetic patients. The previously mentioned studies indicate that a disease known for complications associated with hyperglycemia might have low risks of postoperative complications in the setting of diabetics than in non-diabetics.

SA is the most commonly used technique in conducting anesthesia for women who undergo elective cesarean section because of its low risk of causing complications to the mother and the fetus in comparison with general anesthesia. The outcomes of this research suggests that the use of spinal anesthesia in obstetric patients is favorable because it aids in controlling glucose concentrations perioperatively. This might be of a great positive outcome in decreasing the incidence of the earlier complications caused by hyperglycemia. As a result, these extra benefits favoring spinal anesthesia over general anesthesia should be conducted during patient counseling about cesarean sections.

This study is not without limitation, a relative unequal size of both groups is one of the limitations. Some variables such as body mass index were not studied.

In conclusion, it is noticeable that a considerable surge in mean plasma glucose levels from glucose-check timing with both general anesthesia and spinal anesthesia. The impact of GA on plasma glucose levels was substantially more than the SA, therefore it indicates that the hormonal stress response is significantly more in GA.

This study was conducted according to STROCSS 2019 guideline [30].

## Funding

No funding or sponsorship was received for this study or publication of this article. The article processing charges were funded by the authors.

## Disclosures

There was no conflict of interest to report.

## Compliance with ethics guidelines

This study was approved by Institutional Review Board at Jordan University of Science and Technology. All procedures performed in this study were in accordance with the 1964 Helsinki Declaration and its later amendments or comparable ethical standards.

## Data availability

The dataset generated and analyzed during the current study is available from the corresponding author on reasonable request.

## Informed consent

Written informed consent was obtained from the participant for the publication of the study.

## Author contribution

All authors contributed significantly and in agreement with the content of the article. All authors were involved in project design, data collection, analysis, statistical analysis, data interpretation and writing the manuscript. All authors presented substantial contributions to the article and participated of correction and final approval of the version to be submitted.

## Registration of research studies

researchregistry6532.

## Guarantor

Diab Bani Hani.

## Provenance and peer review

Not commissioned, externally peer-reviewed.
